# A Text-Book of the Diseases of the Ear

**Published:** 1891-04

**Authors:** 


A TEXT-BOOK OF THE DISEASES OF THE EAR. BY DR. JOSEF GRUBER, Professor of Otology in the Imperial Royal University of Vienna, etc. Translated from the Second German edition by special permission of the author, and edited by Edward Law, M. D., C. M., Edin., M. R. C. S., Eng., Surgeon to the London Throat Hospital for Diseases of the Throat, Nose and Ear; and by Coleman Jewell, Lond., M. D., M. R. C. S., Eng. Late Physician and Pathologist to the London Throat Hospital. With 150 illustrations and 70 colored figures on two lithographic plates. Cloth, large 8vo, pp. xxiv.—580. New York. D. Appleton & Co. 1890. Buffalo: Otto Ulbrich.

Eighteen years elapsed between the first and second editions of Gruber's work, the first edition appearing in 1870, and the second in 1888. In the latter we have, therefore, the results of his mature judgment upon observations already made, as well as the record of new investigations. This second edition has been rendered into English by Drs. Law and Jewell, as stated above, and we desire, along with others, to express our obligations to them on the clear and faithful manner in which their task has been accomplished. We appear to be reading an English book, so evenly has the translations been made, and this adds much to the pleasure of our study of it. The first part of the work contains the Anatomy and Physiology of the Organ of Hearing. The anatomical part is exhaustive, and is really a treatise by itself. For reference, we know of nothing more complete—almost too complete we were about to say—to appear in a text-book for students, though for reference, as we have just mentioned, it is invaluable. It occupies 107 pages, while the physiological part is contained within seven pages—in smaller type, however. This great contrast reminds us of Gruber's remark : "Had the results of physiological inquiry in the same department been comparable to that of anatomical investigation, the position of otology at the present day might well have vied with that of the most advanced specialty." This statement should do more than attract our attention; it should stimulate inquiry and experimentation.

The chapters upon the Examinations of Patients are very full, and of great value. The watch test of the hearing power Gruber considers imperfect, but as it is of easy application and of some merit, he gives a few very clear rules for its employment. Of the Estimation of the Hearing Capacity for Speech, he holds rather middle ground between Oscar Wolf, who considers it the most perfect method of testing the hearing power, and Hartmann, who thinks that the test is too complicated to insure accuracy. Of the Modern Methods of Testing the Hearing Power, those of Weber, Rinne, Gelle and Gruber are fully described.

Of the Ability to Hear Better in a Noise (*Hyperacusis Willisii*), this statement is made : "The author has been unable to accept any of the theories hitherto advanced, and he imagines that its cause must be referred to different circumstances, partly pathological, partly physiological, which may happen to coexist in the ear, and bring about a condition favorable to the better conductors of a powerful auditory stimulus."

In the part on Objective Examinations, the author gives full account of the methods of examination of the nose and naso-pharynx. Indeed, throughout the work, Gruber insists upon the importance of these parts as bearing upon troubles with the ear. Thus it is seen that the nose, throat and ear are being associated more and more in the minds of those who are studying the diseases of these organs. That is the only rational and correct division. We regret that the teaching bodies do not so recognize it, and appoint their instructors accordingly. We believe it is sure to come to this. The long existing associations between the eye and ear will be done away with; they have almost no connection in nature, why should they have in practice or in teaching? and the ear, the nose and the throat will be united in one specialty.

For anterior rhinoscopy, Gruber prefers his ear speculum, adapting the size to the case. He also speaks well of the bivalve speculum, but he does not mention Elsberg's trivalve, which, in our opinion, is the best nasal speculum yet invented. Zaufal's instrument is not considered with much favor. He says truly that less can be seen with it than with a large ear speculum, and that it must be introduced with the greatest care, as lesions may easily be caused by the sharp edge of the small end.

In regard to the difficulties of posterior rhinoscopy, he quotes, with approval, the statement of Schwartze, that in seven per cent. of his patients in whom a rhinoscopical examination was desirable, it was absolutely impossible; in twenty-one per cent., only isolated parts could be seen; fifty-five per cent. could be examined with ease; and seventeen per cent. only by much painstaking and quickness of manipulation.

The use of the air douche, with advantages and disadvantages of the various methods employed, receives careful treatment; indeed, nowhere do we remember seeing a more satisfactory consideration of this subject. The author makes a suggestion in regard to limitating the inflation to a single tube that seems of value. The method is as follows: "If the patient bends his head strongly towards one shoulder, the air may be made to enter the middle ear on the side turned away from that shoulder, especially if the nozzle of the air bag be inserted into the nostril of the same side as the ear to be inflated. At any rate, should air enter both ears, more pressure is felt by the patient in the one which is directed upwards." He is in doubt as to the correct explanation of this observation, but we can easily understand its importance in certain cases.

Passing now to the part on Special Diseases, we find the same clear and direct manner of statement, and the same thoroughness observed in the first part. We say this because it is impossible to notice in detail the many subjects here considered. We can only thus express our opinion as to the whole, and, for the rest, present a few things that have attracted us during the study of the work.

Eczema of the External Ear receives some attention, and a point, new to the reviewer, is given in the differentiation between it and diffuse inflammation of the external auditory canal. If there is absence of perforation of the tympanic membrane, the case is probably eczematous. Perforation is generally present in long continued otitis externa diffusa.

A long chapter is devoted to the inflammations of the external auditory canal. These are of two varieties, the *circumscribed* and the *diffused*. In the first, the circumscribed (Furuncle), Gruber uses his medicated gelatine preparations. In the early stage he introduces the "amygdalæ auriums," or "aural ovoids," as they are known in England, of either one-sixth gr. of liquid of opium, or one-twelfth gr. hydrochlorate of morphia. He first washes out the canal with a four per cent. solution of carbolic acid (warm) and the gelatine-almond is then introduced. The effect of these almonds are twofold, anesthetic and antiphlogistic. This treatment is so successful that he no longer practises scarification.

The chapters on Affections of the Membrana Tympani are of special merit, and will be of assistance not only to the new student but to the physician who is familiar with ear diseases. The parts also upon Perforations and Artificial Drums and the chapter on New Formations of the External Ear deserve special mention. The long chapter on Inflammation of the Middle Ear is naturally one that will attract attention. When it is remembered that about two-thirds ($\frac{2}{3}$) of all ear troubles occur in the middle ear, the importance of this chapter is seen at once. It is very exhaustive and requires careful study. Gruber impresses upon us again and again the necessity of treating the diseases of the neighboring parts, viz., the nose, naso-pharynx, pharynx and mouth, including the teeth, if such are found, in every case of middle ear disease. We are reminded here of a recent statement of Goodwillie, to the effect that not every case of nasal disease is followed by disease of the ear, but that in almost every case of middle ear disease will be found some pathological condition of the nose. These two opinions, coming from opposite points of view, so to speak, Gruber being primarily an otologist and Goodwillie having devoted, in the first place, most of his attention to nasal diseases, indicates as strongly as it is possible the truth of the statement above, that these organs must be united in one specialty.

Gruber quotes without disapproval the practice of Killian in using a 20 per cent. solution of menthol in olive oil, to produce contraction of the turbinated bodies. From a large experience with menthol, we are prepared to say that it is not justifiable to use such a percentage in the nose. It is exceedingly painful, even in weak solutions, and must be employed with care. Referring to middle ear disease, with deafness sometimes seen in whole families, the author says: "In such cases the individuals generally possess delicate, easily perspiring skins, and they are often accustomed to regard the deafness which overtakes them, one by one, as an hereditary disease, when it is really due to nothing more than persistently neglected colds." Where there is neuralgia and middle ear catarrh, he says the teeth should always be examined, as often a carious tooth will be found to be the cause of the difficulty.

Want of space prevents a further analysis of this most important work. It will ever remain a monument to the science of otology. It contains in itself and by reference practically all that is known upon the subject today. It points out by indicating what is not yet known, the lines investigation must take in order still to advance the knowledge of the diseases of the ear. It is a work for constant use and will seldom disappoint those who consult its pages.

The publishers, Messrs. Appleton & Co., have presented the book to us in their best manner. The letter press is excellent, but among all the many points of merit in this connection must be mentioned the two colored lithographic plates with their seventy (70) figures of the membranum tympani in health and disease. Seldom have we seen finer work than these plates exhibit.

